# In vitro activity of the antimalarial, antibiotic drugs fosmidomycin and clindamycin against clinical isolates of bacterial bloodstream infections in febrile, hospitalized Ghanaian children

**DOI:** 10.1186/s12936-025-05754-3

**Published:** 2026-01-16

**Authors:** Christoph Pfaffendorf, Sebastian G. Wicha, Elina Petersen, Jürgen May, Miklas Martens, Charity Wiafe Akenten, Ghyslain Mombo-Ngoma, Peter G. Kremsner, Denise Dekker, Michael Ramharter, Johannes Mischlinger

**Affiliations:** 1 Institute of Pharmacy, Department of Clinical Pharmacy, University of Hamburg, Hamburg, Germany; 2 Center for Tropical Medicine, Bernhard Nocht Institute for Tropical Medicine & I Dept. of Medicine University Medical Center Hamburg-Eppendorf, Hamburg, Germany; 3 Infectious Disease Epidemiology, Bernhard Nocht Institute for Tropical Medicine, Hamburg, Germany; 4 German Center for Infection Research (DZIF), Partner Site Hamburg-Lübeck-Borstel-Riems, Hamburg-Lübeck-Borstel-Riems, Bernhard-Nocht-Strasse 74, Hamburg, Germany; 5 Kumasi Centre for Collaborative Research in Tropical Medicine (KCCR), Kumasi, Ghana; 6 Department of Pathobiology, School of Veterinary Medicine, Kwame Nkrumah University of Science and Technology, Kumasi, Ghana; 7 Centre de Recherches Médicales de Lambaréné, Lambaréné, BP 242 Gabon; 8 Department of Implementation Research, Bernhard Nocht Institute for Tropical Medicine, Hamburg, Germany; 9 Institut Für Tropenmedizin, Eberhard-Karls-Universität Tübingen, Wilhelmstraße 27, 72074 Tübingen, Germany; 10 German Center for Infection Research (DZIF), Partner Site, Tübingen, Germany; 11 Research Group One Health Bacteriology, Bernhard Nocht Institute for Tropical Medicine (BNITM), Hamburg, Germany

**Keywords:** Fosmidomycin, Clindamycin, Malaria, Bacterial blood stream infection, Sub-Saharan Africa

## Abstract

**Background:**

The clinical and laboratory distinction between malaria and bacterial blood stream infections in patients with undifferentiated fever remains challenging. Misclassification may result in ineffective treatment with the risk of progression of disease and subsequent morbidity and mortality. A pragmatic solution is the use of multi-disease treatments effective against both malaria and the clinically most relevant bacterial pathogens. Fosmidomycin and clindamycin are two antibiotics that have demonstrated high efficacy in treating malaria. A multi-drug combination treatment of fosmidomycin, clindamycin, and artesunate has been evaluated for the treatment of malaria. To further explore whether fosmidomycin and clindamycin could be used in combined anti-malarial, anti-bacterial broad-spectrum chemotherapeutic treatment, the in vitro anti-bacterial activity of fosmidomycin and clindamycin was assessed against clinically relevant bacterial pathogens responsible for blood stream infections in sub-Saharan African children.

**Methods:**

In vitro drug susceptibility testing was performed using clinical isolates of bacterial bloodstream infections from febrile, hospitalized children in Ghana. Isolates included the most common bacteria responsible for bloodstream infections in sub-Saharan African children, comprising *Klebsiella pneumoniae, Escherichia coli, Streptococcus pneumoniae, Staphylococcus aureus,* and non-typhoidal *Salmonella* (NTS)*.* Minimum Inhibitory Concentrations of fosmidomycin and clindamycin were determined using the agar dilution method.

**Results:**

The findings of in vitro antimicrobial susceptibility testing demonstrated that all tested strains showed susceptibility to at least one of the antibiotics tested. Gram-negative strains demonstrated susceptibility to fosmidomycin (100%) but resistance to clindamycin, while gram-positive strains showed the opposite pattern: susceptibility to clindamycin (100%) but resistance to fosmidomycin.

**Conclusions:**

The results underscore the potential of combining fosmidomycin and clindamycin in antimalarial treatment regimens as a multi-disease treatment strategy, particularly for febrile children. Such an approach would target both *Plasmodium falciparum* and the clinically most relevant bacterial pathogens, addressing one of the most important diagnostic and treatment challenges faced in malaria-endemic regions.

**Supplementary Information:**

The online version contains supplementary material available at 10.1186/s12936-025-05754-3.

## Background

The clinical presentation of *Plasmodium falciparum* malaria is nonspecific and can be difficult to distinguish from febrile infections [1]. Malaria leads to high mortality and morbidity, with the global burden of disease highest in sub-Saharan Africa among young children [2]. Undifferentiated fever in paediatric patients constitutes a diagnostic challenge in regions of high malaria transmission. Rapid diagnostic tests (RDTs) are often used for the detection of *Plasmodium* infections instead of the gold standard microscopy due to operator-friendliness and simplicity of use [2]. Moreover, case management guidelines often follow simple diagnostic and treatment algorithms, in which febrile children with positive malaria RDT results receive treatment with antimalarials [2, 3]. However, since a substantial proportion of residents in highly endemic regions are asymptomatic carriers of *Plasmodium* parasites, other aetiologies for acute febrile illnesses including bacterial blood stream infection are common, despite a positive malaria test [1]. Therefore, an asymptomatic, low-level *Plasmodium* parasitaemia may yield a true positive RDT result, while the patient may suffer from another cause of disease, such as lower respiratory tract infection, pneumonia, urinary tract infection, gastrointestinal infection, or bacterial sepsis. To date, simultaneous diagnostic testing for alternative etiologies of undifferentiated fever such as microbiological testing for bacterial bloodstream infection is often not feasible in resource-limited settings. Therefore, studies have suggested that a substantial proportion of African febrile children in malaria-endemic areas may receive inadequate causative treatment leading to increased morbidity and mortality [1, 4].

A pragmatic and promising approach to this case management dilemma is to conceptualize new multi-disease treatment regimens in which antibiotic and antiparasitic combination therapies are used, covering the most common and clinically relevant infectious disease etiologies of critically ill children with fever [5]. The combination of artesunate with fosmidomycin and clindamycin constitutes an antibiotic-based antimalarial combination therapy with the potential to treat malaria and relevant bacterial pathogens. Previous studies showed high efficacy, and favourable safety and tolerability profiles for these drugs in treating *P. falciparum* malaria [1, 6, 7]. However, to date evidence is lacking whether these drugs may at the same time effectively treat the clinically most relevant bacterial blood stream infections. Furthermore, while the antimicrobial properties of fosmidomycin and clindamycin are well-established in theory, their activity remains untested against bacterial isolates from African pediatric populations, the very patients for whom this dual-purpose therapeutic strategy is intended. To further assess the potential of these drugs in febrile patient management, this study evaluated the in vitro activity of fosmidomycin and clindamycin against clinical isolates of bacterial bloodstream infections from Ghanaian children hospitalized for severe febrile disease.

## Methods

This ancillary study was performed using bacterial blood culture isolates from a prospective, clinical cohort of children hospitalized for acute severe febrile disease, under the age of five years, and residing in a malaria endemic region in the sub-Saharan African country of Ghana. For blood culture collection, 1–3 mL venous blood was collected with at least one of the following inclusion criteria; suspected sepsis, fever (≥ 37.5 °C), or a history of fever within the past 48 h and admitted to Agogo Presbyterian hospital, Ghana. The patient’s blood was drawn into Becton Dickinson (BD) BACTEC® Peds Plus Medium. Blood cultures were processed using a BACTEC® 9050 blood culture system (Becton Dickinson, USA) according to manufacturer’s instructions. For positive blood cultures, aspirated blood culture fluid was Gram stained for preliminary identification and inoculated on Columbia blood-, chocolate-, and MacConkey agar (all Oxoid, Basingstoke, UK). Plates were incubated at 35–37 °C for 18–24 h in normal atmosphere. Bacteria were identified by standard biochemical methods and preserved at -80C using microbanks until transport on dry ice to Germany for further analyses. For this current study, bacterial isolates were used which were stored at -80° C in Germany; they were further cultured and tested for the analyses of this ancillary study. Before testing, the isolates were cultured and stored in broth tubes. The broth tubes were refrigerated and maintained for a maximum of 14 days before subsequent cultivation for testing of minimum inhibitory concentration (MIC).

Representative isolates of the major bacterial pathogens were selected for in vitro antibiotic susceptibility testing [8–10]. These isolates had been collected by the umbrella study between 2010 and 2015. This included *Klebsiella pneumoniae, Escherichia coli, Streptococcus pneumoniae, Staphylococcus aureus,* and non-typhoidal *Salmonella* (NTS). These included ten non-duplicate isolates of *K. pneumoniae*, NTS and *S. aureus*, nine isolates of *E. coli* and 11 isolates of *S. pneumoniae*. All tests were performed in triplicate.

MICs were determined using the agar dilution method as described by the European Committee on Antimicrobial Susceptibility Testing (EUCAST) [11]. Similar to fosfomycin, fosmidomycin yields inconsistent results in broth dilution assays; therefore, agar dilution was preferred for susceptibility testing. To ensure methodological consistency, agar dilution was also used for clindamycin. Plates were prepared with Mueller–Hinton agar (Oxoid Ltd., Basingstoke, UK) supplemented with varying concentrations of clindamycin or fosmidomycin. For fosmidomycin plates, glucose-6-phosphate was added at 25 mg/L to ensure optimal drug activity. Isolates were grown overnight on Mueller–Hinton agar plates. For inoculation, multiple colonies were suspended in 0.9% NaCl to achieve a 0.5 McFarland standard, and 1 μL of this suspension was spotted onto each test plate. Each isolate was tested in triplicate across three individual plates per concentration. Plates were incubated for 18 h at 37 °C in air. The MIC was defined as the lowest concentration which completely inhibits growth. As recommended by EUCAST, single colonies were disregarded as well as a thin haze within the area of the inoculated spot. Growth control plates without antibiotics were included to confirm bacterial viability. Quality control was performed using *E. coli* ATCC 25922 for fosmidomycin and *S. aureus* ATCC 29213 for clindamycin (EUCAST expected MIC: 0.125 mg/L). While no published MIC values exist for *E. coli* ATCC 25922 with fosmidomycin, this strain was included as a quality control to ensure consistency across all fosmidomycin testing [11, 12]. *S. pneumoniae* was tested on Mueller–Hinton-agar (Oxoid) supplemented with 5% defibrinated horse blood and 20 mg/L β-Nicotinamide adenine dinucleotide (β-NAD), which is the medium recommended by EUCAST [13, 14].

For clindamycin, MICs were assessed according to the EUCAST cut-off values [15]. To date, for fosmidomycin, no established EUCAST reference values have been defined. In vivo studies demonstrated a protective effect of fosmidomycin in mice infected with *E. coli*, exhibiting a MIC of up to 50 mg/L, and in *K. pneumoniae*, with an MIC of 6.25 mg/L [16]. Considering the mean trough concentration of fosmidomycin observed at 1.06 mg/L, a threshold was established for antibiotic susceptibility, defining strains with MICs 1 mg/L or lower as susceptible [7]. This is, however, an exploratory threshold for research purposes and should not be interpreted as a clinical breakpoint.

## Ethics statement

In Ghana, The Committee on Human Research, Publications and Ethics, School of Medical Science, Kwame Nkrumah University of Science and Technology in Kumasi, Ghana, approved this study (No. CHRPE/AP/674/19). In Germany approval was granted by the Ethikkommission der Ärztekammer Hamburg (No. PV5664). Noagoya protocols were approved by the local respective focal point. Study participants were informed about the purpose of this study and the study procedures. Written informed consent was obtained before enrolment from the child’s parent or guardian.

## Results

Fosmidomycin did not exhibit antibacterial activity against the Gram-positive bacteria *S. aureus* and *S. pneumoniae*. For Gram-negative bacteria, the MIC of fosmidomycin was 0.5–1 mg/L for *K. pneumoniae,* and 0.125 to 0.25 mg/L for *E. coli* and *S. enterica.* The quality control strain *E. coli* ATCC 25922 consistently showed an MIC of 1 mg/L.

Clindamycin showed high activity against Gram-positive bacteria, with low MIC values. The growth of *S. aureus* was inhibited at clindamycin concentrations between 0.03–0.06 mg/L, with the EUCAST break-point set at 0.25 mg/L. For the seven *S. pneumoniae*, MICs were even lower, at < 0.016 mg/L, compared to the EUCAST breakpoint of < 0.026 mg/L [15]. Four strains of *S. pneumoniae* could not be cultured for clindamycin testing. For the inhibition of Gram-negative bacteria, high MICs were observed for clindamycin, ranging from < 128–128 mg/L for *E. coli* and *K. pneumoniae* to 128–256 mg/L for *S. enterica.* The quality control stain *S. aureus* ATC 29213 consistently showed the expected MIC of 0.125 mg/L. MIC results are shown in Table 1. Full results for each isolate are shown in the supplement (S1).

**Table 1:** Minimum inhibitory concentrations [mg/L] for fosmidomycin and clindamycin in clinical isolates of selected Gram-negative and Gram-positive bacteria

Organism	Antibiotic	Breakpoint [mg/L]	Isolates [n]	MIC50 [mg/L]	MIC90 [mg/L]	Susceptible [%]
Gram-Negative						
*Escherichia coli*	Clindamycin	n.a	9	128.000	128.00	0.0
*Escherichia coli*	Fosmidomycin	1.0^*^	9	0.250	0.25	100.0
*Klebsiella pneumoniae*	Clindamycin	n.a	10	128.000	128.00	0.0
*Klebsiella pneumoniae*	Fosmidomycin	1.0^*^	10	1.000	1.00	100.0
Non-typhoidal Salmonella (NTS)	Clindamycin	n.a	10	256.000	256.00	0.0
Non-typhoidal Salmonella (NTS)	Fosmidomycin	1.0^*^	10	0.125	0.25	100.0
Gram-Positive						
*Staphylococcus aureus*	Clindamycin	0.250	10	0.06	0.06	100.0
*Staphylococcus aureus*	Fosmidomycin	1.0^*^	10	> 512	> 512	0.0
*Streptococcus pneumoniae*	Clindamycin	0.026	11	< 0.016	< 0.016	100
*Streptococcus pneumoniae*	Fosmidomycin	1.0^*^	11	> 32	> 32	0.0

Clindamycin is not active against Gram-negative bacteria. Therefore, susceptibility breakpoints are not established for Gram-negative species, and they are marked as n.a. (Not Applicable)

*The breakpoint for fosmidomycin is based on assumptions and should not be interpreted as a clinical breakpoint

## Discussion

This study evaluated MICs of two antibiotic drugs with anti-malarial activity. The findings demonstrate that all bacterial strains tested were susceptible to at least one of the antibiotics. The activities of the two drugs are complementary, with fosmidomycin being effective against Gram-negative strains and clindamycin showing activity against Gram-positive strains. Fosmidomycin and clindamycin have been of particular interest because of their efficacy, safety, and tolerability in treating malaria in combination with an artemisinin-derivative, which has been demonstrated in several studies using varying combination partners [17, 18]. Recently, a randomized-controlled phase II clinical study was published which investigated the triple combination therapy artesunate/fosmidomycin/clindamycin in treating falciparum malaria (PACTR registration number: PACTR202008909968293) [19]. The study reported an excellent anti-malarial efficacy with a favourable safety and tolerability profile, thereby confirming the potential of artesunate/fosmidomycin/clindamycin and paving the way for future, larger phase III clinical trials of this triple combination treatment. However, there is no published study that investigated the efficacy of fosmidomycin and/or clindamycin to treat critically ill febrile children in malaria-endemic areas from bacterial infections. Therefore, this current in vitro study provides first important data to evaluate the antibacterial activity on the most common, clinically relevant bacterial pathogens causing critical bloodstream infections in sub-Saharan African children.

In this study, clindamycin was reliable at inhibiting the growth of the Gram-positive bacteria *S. aureus* and *S. pneumoniae*, while not being efficacious against the Gram-negative bacteria *K. pneumoniae*, *E. coli* and *Salmonella enterica.* These results are concordant with susceptibilities reported by the EUCAST [15]. For one *K. pneumoniae* and three *E. coli* strains, the MICs were found to be below the tested range. Since the majority of the strains demonstrated resistance to clindamycin, the MICs for these strains were not retested. On the other hand, fosmidomycin was active against the Gram-negative bacteria, as suggested by the low MIC values relating to Gram-negative isolates tested in this study. Fosmidomycin had no effect on the Gram-positive bacteria as demonstrated by the MICs > 32 and > 256. These findings were to be expected, as both *S. aureus* and *S. pneumoniae* do not rely on the methylerythritol phosphate (MEP) pathway and thus are lacking the target of fosmidomycin [20]. These data indicate that, at least in theory, fosmidomycin and clindamycin when administered in combination may be able to cover both infections with Gram-positive and Gram-negative bacteria, which can cause serious clinical disease. Since there are currently no available EUCAST breakpoints for fosmidomycin, there is uncertainty concerning the in vivo susceptibility. However, clinically observed concentrations in patients with acute uncomplicated falciparum malaria treated with oral fosmidomycin reached mean trough concentrations of 1 mg/L and therefore show a time above the MIC for the tested Gram-negative bacteria close to 100% [7]. It should be noted, however, that fosmidomycin susceptibility results require cautious interpretation, as the defined threshold is based on theoretical assumptions rather than clinical outcome data. This study is limited by the modest sample size (9–11 isolates per species), which may not capture the full diversity of resistance patterns in the region. Furthermore, not all *S. pneumoniae* isolates could be cultured, which could introduce potential bias into the study. Therefore, further research, particularly within the framework of clinical randomized controlled trials assessing microbiological endpoints, will certainly be crucial to further elucidate the role of fosmidomycin in treating Gram-negative bacterial blood stream infections. The complementary activity observed in this study aligns with known resistance mechanisms. The inactivity of clindamycin against Enterobacterales reflects intrinsic resistance due to poor outer membrane penetration and active efflux in Gram-negative bacteria [21]. Furthermore, fosmidomycin requires active transport via the glycerol-3-phosphate (GlpT) and hexose phosphate (uhpT) transporters, which are present in Enterobacterales but absent in Gram-positive bacteria [22]. Additionally, *S. aureus* and *S. pneumoniae* lack the MEP pathway for isoprenoid synthesis, utilizing the mevalonate pathway instead, explaining their fosmidomycin resistance. [17, 23]. These mechanisms have clinical implications. While the combination theoretically covers both Gram-positive and Gram-negative pathogens, the transport-dependent uptake of fosmidomycin may result in variable in vivo activity, particularly under conditions affecting transporter expression [24]. Resistance can rapidly develop through glpT or uhpT mutations. Coverage gaps remain for organisms with dual resistance, such as *Enterococcus* species lacking both clindamycin susceptibility and functional fosmidomycin transporters [25].

This study intended to provide evidence to further aid in the development of a broad-spectrum treatment with combined anti-malarial and anti-bacterial activity with the ultimate aim to address a current case management dilemma. This dilemma results from the phenomenon that many children particularly in high malaria transmission regions are asymptomatic *Plasmodium* parasite carriers, which leads to the paradoxical situation in which a positive malaria diagnostic test result would—even though parasitologically true positive—prompt clinicians to mismanage non-malarial febrile disease episodes by prescribing anti-malarials. This diagnostic dilemma is further aggravated by the impossibility to clinically distinguish severe malaria from bacterial sepsis in critically ill children. Therefore, studies have indicated that a substantial proportion of febrile African children might not receive adequate fever management. This case management dilemma could probably be most efficiently addressed by improving differential diagnosis capabilities across sub-Saharan African settings to rule out non-malarial pathogens and coinfections in febrile patients. However, this is hindered by the limited possibilities of bacterial culture methods in many sub-Saharan settings. The advent of molecular, multiplex diagnostics which allow for the detection of several bacterial (and viral) targets at once could also be helpful. Yet, to date, this technology is mostly limited to high-resource settings and is therefore unlikely to have a strong impact in the short-to-mid-term future. A broad-spectrum treatment for critically ill children in malaria-endemic regions with activity against most relevant infectious fever causes would, therefore, pose a crude, yet pragmatic solution.

In addition to bacteria and *P. falciparum*, other pathogens, especially viruses, also cause a significant proportion of serious and febrile infections in malaria-endemic regions [26]. As a result, there is a potential for misuse and overuse of antibiotics, leading to increased antimicrobial resistance (AMR). Given that the mis-and overuse of antibiotics is one of the leading causes for the spread of resistance caution is necessitated when introducing a broad-spectrum treatment with combined anti-malarial, anti-bacterial activity. One measure to halt bacterial resistance development would be to limit the use of an fosmidomycin-clindamycin-containing regimen (e.g. artesunate-fosmidomycin-clindamycin) to critically ill children, as opposed to children with uncomplicated malaria or bacterial diseases. Such adequate antibiotic stewardship would—at least theoretically—warrant safe and sustainable long-term use [27]. Furthermore, wherever possible anti-microbial chemotherapy should always be preceded by an adequate diagnostic rather using broad-spectrum treatments. In this regard it was reported that antibiotic overuse can be decreased by improving differential diagnosis in febrile patients suspected of having malaria, to rule out other pathogens or coinfections [28].

As an in vitro study, this research cannot address all potential clinical implications, as there needs to be clinical studies assessing the efficacy of a fosmidomycin-clindamycin combination treatment on bacterial infections. Also, this study focused on selected bacteria and strains and the sample size was rather small thus, including more strains or other common bacteria in malaria-endemic regions is an important aspect for future studies. However, it is likely that this study addressed the most clinically relevant bacterial strains relevant in sub-Saharan African hospitalized, febrile children, as also indicated by the to date largest comprehensive study which systematically compiled the prevalence of bacterial species being responsible for community-acquired blood stream infections in Africa [8]. Lastly, substances with anti-viral activity were not considered in the rationale of covering most important fever causes of critically ill children. However, first, given limited treatment possibilities for tropical virus diseases and second, that there is no anti-viral treatment with simultaneous anti-malarial activity it is believed that this does not constitute a major limitation related to the current proposition of a fosmidomycin-clindamycin-containing broad-spectrum treatment.

## Conclusions

Fosmidomycin and clindamycin demonstrate high activity against clinical isolates of bacterial blood stream infections in this in vitro study. Based on these data, it seems promising to use the two anti-malarial antibiotics fosmidomycin and clindamycin with artesunate (i.e. the backbone of contemporary first line treatment of uncomplicated and severe malaria) in a triple combination therapy. Doing so may lead to the development of an effective drug regimen to treat acute malaria and bacterial bloodstream infections in African children ultimately posing a solution of the current case management dilemma of febrile children in malaria-endemic countries. While the results are promising, further research including synergy testing, time kill experiments, studies using dynamic hollow fiber systems, in vivo investigations as well as PK-PD modelling is necessary to strengthen the evidence for clinical applicability.

## Supplementary Information

Additional file 1.

## Data Availability

All data generated or analysed during this study are included in this published article.

## Abbreviations

CFU: Colony-forming units
EUCAST: European Committee on Antimicrobial Susceptibility Testing
iCCM: Integrated community case management
MIC: Minimum inhibitory concentration
NPV: Negative predictive value
PPV: Positive predictive value
RDT: Rapid diagnostic test

## References

[1] Mischlinger J, Dudek V, Ramharter M. Predictive performance of rapid diagnostic tests for falciparum malaria and its modeled impact on integrated community case management of malaria in sub-Saharan African febrile children. Clin Infect Dis. 2021;73:e1158–67. 33506270 10.1093/cid/ciaa1942 PMC8423504

[2] WHO. Guidelines for malaria, 30 November 2024. Geneva, World Health Organization, 2024.

[3] WHO. Community-based reduction of malaria transmission - Consultation Report. Geneva, World Health Organization, 2012.

[4] Dalrymple U, Cameron E, Bhatt S, Weiss DJ, Gupta S, Gething PW. Quantifying the contribution of *Plasmodium falciparum* malaria to febrile illness amongst African children. Elife. 2017;6:e29198. 29034876 10.7554/eLife.29198 PMC5665646

[5] Noedl H. ABC - antibiotics-based combinations for the treatment of severe malaria? Trends Parasitol. 2009;25:540–4. 19819757 10.1016/j.pt.2009.09.001

[6] Oyakhirome S, Issifou S, Pongratz P, Barondi F, Ramharter M, Kun JF, et al. Randomized controlled trial of fosmidomycin-clindamycin versus sulfadoxine-pyrimethamine in the treatment of *Plasmodium falciparum* nalaria. Antimicrob Agents Chemother. 2007;51:1869–71. 17325227 10.1128/AAC.01448-06 PMC1855537

[7] Na-Bangchang K, Ruengweerayut R, Karbwang J, Chauemung A, Hutchinson D. Pharmacokinetics and pharmacodynamics of fosmidomycin monotherapy and combination therapy with clindamycin in the treatment of multidrug resistant falciparum malaria. Malar J. 2007;6:70. 17531088 10.1186/1475-2875-6-70 PMC1896174

[8] Reddy EA, Shaw AV, Crump JA. Community-acquired bloodstream infections in Africa: a systematic review and meta-analysis. Lancet Infect Dis. 2010;10:417–32. 20510282 10.1016/S1473-3099(10)70072-4 PMC3168734

[9] Hogan B, Eibach D, Krumkamp R, Sarpong N, Dekker D, Kreuels B, et al. Malaria coinfections in febrile pediatric inpatients: a hospital-based study from Ghana. Clin Infect Dis. 2018;66:1838–45. 29408951 10.1093/cid/cix1120 PMC5982794

[10] Wilairatana P, Mala W, Masangkay FR, Kotepui KU, Kotepui M. The prevalence of malaria and bacteremia co-infections among febrile patients: a systematic review and meta-analysis. Trop Med Infect Dis. 2022;7:243. 36136654 10.3390/tropicalmed7090243 PMC9503679

[11] EUCAST Definitive Document E.DEF 3.1, June 2000: Determination of minimum inhibitory concentrations (MICs) of antibacterial agents by agar dilution. Clin Microbiol Infect. 2000;6:509–15. 10.1046/j.1469-0691.2000.00142.x 11168187

[12] Wiegand I, Hilpert K, Hancock RE. Agar and broth dilution methods to determine the minimal inhibitory concentration (MIC) of antimicrobial substances. Nat Protoc. 2008;3:163–75. 18274517 10.1038/nprot.2007.521

[13] Kowalska-Krochmal B, Dudek-Wicher R. The minimum inhibitory concentration of antibiotics: methods, interpretation, clinical relevance. Pathogens. 2021;10:165. 33557078 10.3390/pathogens10020165 PMC7913839

[14] EUCAST. Media preparation for EUCAST disk diffusion testing and for determination of MIC values by the broth microdilution method. Version 7.0, 2022. European Committee on Antimicrobial Susceptibility Testing 2022. Available from: https://www.eucast.org.

[15] The European Committee on Antimicrobial Susceptibility Testing. Breakpoint tables for interpretation of MICs and zone diameters. 2024.

[16] Mine Y, Kamimura T, Nonoyama S, Nishida M, Goto S, Kuwahara S. In vitro and in vivo antibacterial activities of FR-31564, a new phosphonic acid antibiotic. J Antibiot (Tokyo). 1980;33:36–43. 7372548 10.7164/antibiotics.33.36

[17] Fernandes JF, Lell B, Agnandji ST, Obiang RM, Bassat Q, Kremsner PG, et al. Fosmidomycin as an antimalarial drug: a meta-analysis of clinical trials. Future Microbiol. 2015;10:1375–90. 26228767 10.2217/FMB.15.60

[18] Ramharter M, Oyakhirome S, Klein Klouwenberg P, Adegnika AA, Agnandji ST, Missinou MA, et al. Artesunate-clindamycin versus quinine-clindamycin in the treatment of *Plasmodium falciparum* malaria: a randomized controlled trial. Clin Infect Dis. 2005;40:1777–84. 15909266 10.1086/430309

[19] Agobé JCD, Maïga-Ascofaré O, Adegnika AA, Pfaffendorf C, Boaheng JM, Edoa JR, et al. Artesunate/pyronaridine-atovaquone/proguanil and artesunate-fosmidomycin-clindamycin compared to standard artesunate/pyronaridine for the treatment of uncomplicated malaria: a phase II randomised controlled clinical trial in Gabon and Ghana [Accepted for publication on 01/SEP/2025]. Lancet Microbe. 2025.

[20] Eberl M, Hintz M, Reichenberg A, Kollas AK, Wiesner J, Jomaa H. Microbial isoprenoid biosynthesis and human gammadelta T cell activation. FEBS Lett. 2003;544:4–10. 12782281 10.1016/s0014-5793(03)00483-6

[21] Leclercq R. Mechanisms of resistance to macrolides and lincosamides: nature of the resistance elements and their clinical implications. Clin Infect Dis. 2002;34:482–92. 11797175 10.1086/324626

[22] Sakamoto Y, Furukawa S, Ogihara H, Yamasaki M. Fosmidomycin resistance in adenylate cyclase deficient (cya) mutants of *Escherichia coli*. Biosci Biotechnol Biochem. 2003;67:2030–3. 14519998 10.1271/bbb.67.2030

[23] Wilding EI, Brown JR, Bryant AP, Chalker AF, Holmes DJ, Ingraham KA, et al. Identification, evolution, and essentiality of the mevalonate pathway for isopentenyl diphosphate biosynthesis in gram-positive cocci. J Bacteriol. 2000;182:4319–27. 10894743 10.1128/jb.182.15.4319-4327.2000 PMC101949

[24] Armstrong CM, Meyers DJ, Imlay LS, Freel Meyers C, Odom AR. Resistance to the antimicrobial agent fosmidomycin and an FR900098 prodrug through mutations in the deoxyxylulose phosphate reductoisomerase gene (dxr). Antimicrob Agents Chemother. 2015;59:5511–9. 26124156 10.1128/AAC.00602-15 PMC4538460

[25] Miller WR, Munita JM, Arias CA. Mechanisms of antibiotic resistance in enterococci. Expert Rev Anti-Infect Ther. 2014;12:1221–36. 25199988 10.1586/14787210.2014.956092 PMC4433168

[26] Kabore B, Post A, Lompo P, Bognini JD, Diallo S, Kam BTD, et al. Aetiology of acute febrile illness in children in a high malaria transmission area in West Africa. Clin Microbiol Infect. 2021;27:590–6. 32505586 10.1016/j.cmi.2020.05.029

[27] Kariuki S, Kering K, Wairimu C, Onsare R, Mbae C. Antimicrobial resistance rates and surveillance in sub-Saharan Africa: where are we now? Infect Drug Resist. 2022;15:3589–609. 35837538 10.2147/IDR.S342753 PMC9273632

[28] Tagoe JNA, Yeboah C, Behene E, Kumordjie S, Nimo-Paintsil S, Attram N, et al. Coinfection of malaria and bacterial pathogens among acute febrile patients in selected clinics in Ghana. Am J Trop Med Hyg. 2023;109:1036–46. 37748764 10.4269/ajtmh.23-0099 PMC10622490

