# Supplementary material for: In vitro activity of the antimalarial, antibiotic drugs fosmidomycin and clindamycin against clinical isolates of bacterial bloodstream infections in febrile, hospitalized Ghanaian children
Source: Malar J. 2026 Jan 16;25:61. doi: 10.1186/s12936-025-05754-3 (PMC12849676; doi:10.1186/s12936-025-05754-3)
Supplement: Supplementary file 1 — Additional file 1. [file 12936_2025_5754_MOESM1_ESM.docx]

**S1** Raw Results of the MIC tests

# Raw Data: All Replicates

## Fosmidomycin - All Replicates

| Organism | Isolate_ID | Antibiotic | Rep1 | Rep2 | Rep3 |
| --- | --- | --- | --- | --- | --- |
| *Escherichia coli* | Iso04645 | Fosmidomycin | 0.125 | 0.125 | 0.125 |
| *Escherichia coli* | Iso04646 | Fosmidomycin | 0.125 | 0.25 | 0.25 |
| *Escherichia coli* | Iso04647 | Fosmidomycin | 0.125 | 0.25 | 0.25 |
| *Escherichia coli* | Iso04648 | Fosmidomycin | 0.125 | 0.125 | 0.25 |
| *Escherichia coli* | Iso04650 | Fosmidomycin | 0.25 | 0.25 | 0.25 |
| *Escherichia coli* | Iso04651 | Fosmidomycin | 0.125 | 0.125 | 0.125 |
| *Escherichia coli* | Iso04652 | Fosmidomycin | 0.25 | 0.25 | 0.25 |
| *Escherichia coli* | Iso04653 | Fosmidomycin | 0.125 | 0.125 | 0.125 |
| *Escherichia coli* | Iso04654 | Fosmidomycin | 0.25 | 0.25 | 0.25 |
| *Klebsiella pneumoniae* | Iso02322 | Fosmidomycin | 0.5 | 1 | 0.5 |
| *Klebsiella pneumoniae* | Iso02323 | Fosmidomycin | 1 | 1 | 0.5 |
| *Klebsiella pneumoniae* | Iso02324 | Fosmidomycin | 1 | 0.5 | 1 |
| *Klebsiella pneumoniae* | Iso02325 | Fosmidomycin | 0.5 | 0.5 | 1 |
| *Klebsiella pneumoniae* | Iso02326 | Fosmidomycin | 1 | 1 | 0.5 |
| *Klebsiella pneumoniae* | Iso02327 | Fosmidomycin | 1 | 0.5 | 1 |
| *Klebsiella pneumoniae* | Iso02328 | Fosmidomycin | 1 | 0.5 | 1 |
| *Klebsiella pneumoniae* | Iso02329 | Fosmidomycin | 0.5 | 1 | 0.5 |
| *Klebsiella pneumoniae* | Iso02330 | Fosmidomycin | 1 | 1 | 0.5 |
| *Klebsiella pneumoniae* | Iso02331 | Fosmidomycin | 0.5 | 1 | 1 |
| Non-typhoidal Salmonella (NTS) | Iso04559 | Fosmidomycin | 0.25 | 0.25 | 0.25 |
| Non-typhoidal Salmonella (NTS) | Iso04560 | Fosmidomycin | 0.25 | 0.25 | 0.25 |
| Non-typhoidal Salmonella (NTS) | Iso04561 | Fosmidomycin | 0.125 | 0.125 | 0.125 |
| Non-typhoidal Salmonella (NTS) | Iso04562 | Fosmidomycin | 0.125 | 0.125 | 0.125 |
| Non-typhoidal Salmonella (NTS) | Iso04563 | Fosmidomycin | 0.125 | 0.125 | 0.125 |
| Non-typhoidal Salmonella (NTS) | Iso04564 | Fosmidomycin | 0.125 | 0.125 | 0.125 |
| Non-typhoidal Salmonella (NTS) | Iso04565 | Fosmidomycin | 0.125 | 0.125 | 0.125 |
| Non-typhoidal Salmonella (NTS) | Iso04566 | Fosmidomycin | 0.125 | 0.125 | 0.125 |
| Non-typhoidal Salmonella (NTS) | Iso04567 | Fosmidomycin | 0.125 | 0.125 | 0.125 |
| Non-typhoidal Salmonella (NTS) | Iso04568 | Fosmidomycin | 0.125 | 0.125 | 0.125 |
| *Staphylococcus aureus* | Iso00662 | Fosmidomycin | >512 | >512 | >512 |
| *Staphylococcus aureus* | Iso00663 | Fosmidomycin | >512 | >512 | >512 |
| *Staphylococcus aureus* | Iso00664 | Fosmidomycin | >512 | >512 | >512 |
| *Staphylococcus aureus* | Iso00686 | Fosmidomycin | >512 | >512 | >512 |
| *Staphylococcus aureus* | Iso00688 | Fosmidomycin | >512 | >512 | >512 |
| *Staphylococcus aureus* | Iso00690 | Fosmidomycin | >512 | >512 | >512 |
| *Staphylococcus aureus* | Iso00691 | Fosmidomycin | >512 | >512 | >512 |
| *Staphylococcus aureus* | Iso00692 | Fosmidomycin | >512 | >512 | >512 |
| *Staphylococcus aureus* | Iso00693 | Fosmidomycin | >512 | >512 | >512 |
| *Staphylococcus aureus* | Iso00694 | Fosmidomycin | >512 | >512 | >512 |
| *Streptococcus pneumoniae* | 114588 | Fosmidomycin | >32 | >32 | >32 |
| *Streptococcus pneumoniae* | 115429 | Fosmidomycin | >32 | >32 | >32 |
| *Streptococcus pneumoniae* | 115658 | Fosmidomycin | >32 | >32 | >32 |
| *Streptococcus pneumoniae* | 500701 | Fosmidomycin | >32 | >32 | >32 |
| *Streptococcus pneumoniae* | 500967 | Fosmidomycin | >32 | >32 | >32 |
| *Streptococcus pneumoniae* | Iso04657 | Fosmidomycin | >32 | >32 | >32 |
| *Streptococcus pneumoniae* | Iso04658 | Fosmidomycin | >32 | >32 | >32 |
| *Streptococcus pneumoniae* | Iso04659 | Fosmidomycin | >32 | >32 | >32 |
| *Streptococcus pneumoniae* | Iso04660 | Fosmidomycin | >32 | >32 | >32 |
| *Streptococcus pneumoniae* | Iso04662 | Fosmidomycin | >32 | >32 | >32 |
| *Streptococcus pneumoniae* | Iso04663 | Fosmidomycin | >32 | >32 | >32 |

## Clindamycin - All Replicates

| Organism | Isolate_ID | Antibiotic | Rep1 | Rep2 | Rep3 |
| --- | --- | --- | --- | --- | --- |
| *Escherichia coli* | Iso04645 | Clindamycin | 128 | 128 | 128 |
| *Escherichia coli* | Iso04646 | Clindamycin | <128 | <128 | <128 |
| *Escherichia coli* | Iso04647 | Clindamycin | 128 | 128 | 128 |
| *Escherichia coli* | Iso04648 | Clindamycin | 128 | 128 | 128 |
| *Escherichia coli* | Iso04650 | Clindamycin | 128 | 128 | 128 |
| *Escherichia coli* | Iso04651 | Clindamycin | <128 | <128 | <128 |
| *Escherichia coli* | Iso04652 | Clindamycin | 128 | 128 | 128 |
| *Escherichia coli* | Iso04653 | Clindamycin | <128 | <128 | <128 |
| *Escherichia coli* | Iso04654 | Clindamycin | 128 | 128 | 128 |
| *Klebsiella pneumoniae* | Iso02322 | Clindamycin | 128 | 128 | 128 |
| *Klebsiella pneumoniae* | Iso02323 | Clindamycin | 128 | 128 | 128 |
| *Klebsiella pneumoniae* | Iso02324 | Clindamycin | <128 | <128 | <128 |
| *Klebsiella pneumoniae* | Iso02325 | Clindamycin | 128 | 128 | 128 |
| *Klebsiella pneumoniae* | Iso02326 | Clindamycin | 128 | 128 | 128 |
| *Klebsiella pneumoniae* | Iso02327 | Clindamycin | 128 | 128 | 128 |
| *Klebsiella pneumoniae* | Iso02328 | Clindamycin | 128 | 128 | 128 |
| *Klebsiella pneumoniae* | Iso02329 | Clindamycin | 128 | 128 | 128 |
| *Klebsiella pneumoniae* | Iso02330 | Clindamycin | 128 | 128 | 128 |
| *Klebsiella pneumoniae* | Iso02331 | Clindamycin | 128 | 128 | 128 |
| Non-typhoidal Salmonella (NTS) | Iso04559 | Clindamycin | 256 | 256 | 256 |
| Non-typhoidal Salmonella (NTS) | Iso04560 | Clindamycin | 256 | 256 | 256 |
| Non-typhoidal Salmonella (NTS) | Iso04561 | Clindamycin | 256 | 256 | 256 |
| Non-typhoidal Salmonella (NTS) | Iso04562 | Clindamycin | 128 | 128 | 128 |
| Non-typhoidal Salmonella (NTS) | Iso04563 | Clindamycin | 256 | 256 | 256 |
| Non-typhoidal Salmonella (NTS) | Iso04564 | Clindamycin | 256 | 256 | 256 |
| Non-typhoidal Salmonella (NTS) | Iso04565 | Clindamycin | 256 | 256 | 256 |
| Non-typhoidal Salmonella (NTS) | Iso04566 | Clindamycin | 256 | 256 | 256 |
| Non-typhoidal Salmonella (NTS) | Iso04567 | Clindamycin | 256 | 256 | 256 |
| Non-typhoidal Salmonella (NTS) | Iso04568 | Clindamycin | 128 | 128 | 128 |
| *Staphylococcus aureus* | Iso00662 | Clindamycin | 0.03 | 0.03 | 0.03 |
| *Staphylococcus aureus* | Iso00663 | Clindamycin | 0.06 | 0.06 | 0.06 |
| *Staphylococcus aureus* | Iso00664 | Clindamycin | 0.03 | 0.03 | 0.03 |
| *Staphylococcus aureus* | Iso00686 | Clindamycin | 0.03 | 0.03 | 0.03 |
| *Staphylococcus aureus* | Iso00688 | Clindamycin | 0.06 | 0.06 | 0.06 |
| *Staphylococcus aureus* | Iso00690 | Clindamycin | 0.06 | 0.06 | 0.06 |
| *Staphylococcus aureus* | Iso00691 | Clindamycin | 0.06 | 0.06 | 0.06 |
| *Staphylococcus aureus* | Iso00692 | Clindamycin | 0.06 | 0.06 | 0.06 |
| *Staphylococcus aureus* | Iso00693 | Clindamycin | 0.06 | 0.06 | 0.06 |
| *Staphylococcus aureus* | Iso00694 | Clindamycin | 0.06 | 0.06 | 0.06 |
| *Streptococcus pneumoniae* | 114588 | Clindamycin |  |  |  |
| *Streptococcus pneumoniae* | 115429 | Clindamycin | <0.016 | <0.016 | <0.016 |
| *Streptococcus pneumoniae* | 115658 | Clindamycin | <0.016 | <0.016 | <0.016 |
| *Streptococcus pneumoniae* | 500701 | Clindamycin |  |  |  |
| *Streptococcus pneumoniae* | 500967 | Clindamycin |  |  |  |
| *Streptococcus pneumoniae* | Iso04657 | Clindamycin | <0.016 | <0.016 | <0.016 |
| *Streptococcus pneumoniae* | Iso04658 | Clindamycin | <0.016 | <0.016 | <0.016 |
| *Streptococcus pneumoniae* | Iso04659 | Clindamycin | <0.016 | <0.016 | <0.016 |
| *Streptococcus pneumoniae* | Iso04660 | Clindamycin | <0.016 | <0.016 | <0.016 |
| *Streptococcus pneumoniae* | Iso04662 | Clindamycin |  |  |  |
| *Streptococcus pneumoniae* | Iso04663 | Clindamycin | <0.016 | <0.016 | <0.016 |
